# Treatment of steroid-induced osteonecrosis of the femoral head using porous Se@SiO_2_ nanocomposites to suppress reactive oxygen species

**DOI:** 10.1038/srep43914

**Published:** 2017-03-03

**Authors:** Guoying Deng, Kerun Niu, Feng Zhou, Buxiao Li, Yingjie Kang, Xijian Liu, Junqing Hu, Bo Li, Qiugen Wang, Chengqing Yi, Qian Wang

**Affiliations:** 1 Trauma Center, Shanghai General Hospital, Shanghai Jiaotong University School of Medicine, 650 Xin Songjiang Road, Shanghai 201620, P.R. China; 2 Department of Orthopedics, Shanghai Bone Tumor Institute, Shanghai General Hospital of Nanjing Medical University, Shanghai 200080, P.R. China; 3 Department of Radiology, Shuguang Hospital, Shanghai University of Traditional Chinese Medicine. No. 528, Zhangheng Road, Shanghai 201203, P.R. China; 4 College of Chemistry and Chemical Engineering, Shanghai University of Engineering Science, Shanghai, 201620, P.R. China; 5 State Key Laboratory for Modification of Chemical Fibers and Polymer Materials, College of Materials Science and Engineering, Donghua University, Shanghai 201620, P.R. China; 6 State Key Laboratory of High Performance Ceramics and Superfine Microstructure, Shanghai Institute of Ceramics, Chinese Academy of Sciences, No. 1295 Dingxi Road, Shanghai 200050, People’s Republic of China; 7 Department of Orthopedics, Shanghai Bone Tumor Institute, Shanghai General Hospital, Shanghai Jiao Tong University School of Medicine, No. 100 Haining Road, Shanghai 200080, P.R. China

## Abstract

Reducing oxidative stress (ROS) have been demonstrated effective for steroid-induced osteonecrosis of the femoral head (steroid-induced ONFH). Selenium (Se) plays an important role in suppressing oxidative stress and has huge potential in ONFH treatments. However the Se has a narrow margin between beneficial and toxic effects which make it hard for therapy use *in vivo*. In order to make the deficiency up, a control release of Se (Se@SiO_2_) were realized by nanotechnology modification. Porous Se@SiO_2_ nanocomposites have favorable biocompatibility and can reduced the ROS damage effectively. *In vitro*, the cck-8 analysis, terminal dexynucleotidyl transferase (TdT)-mediated dUTP nick end labeling (TUNEL) stain and flow cytometry analysis showed rare negative influence by porous Se@SiO_2_ nanocomposites but significantly protective effect against H_2_O_2_ by reducing ROS level (detected by DCFH-DA). *In vivo*, the biosafety of porous Se@SiO_2_ nanocomposites were confirmed by the serum biochemistry, the ROS level in serum were significantly reduced and the curative effect were confirmed by Micro CT scan, serum Elisa assay (inflammatory factors), Western blotting (quantitative measurement of ONFH) and HE staining. It is expected that the porous Se@SiO_2_ nanocomposites may prevent steroid-induced ONFH by reducing oxidative stress.

Steroid as an irreplaceable medicaments is used to treat diseases including rheumatoid arthritis, systemic lupus erythematosus, acute lymphoblastic leukaemia and so on. However, steroid-induced osteonecrosis of the femoral head (ONFH) has been one of the most serious diseases for orthopedists, and a hip replacement is the only treatment option at the terminal stage^1^. Epidemiology studies in East Asia show that 47.4% of all cases diagnosed as non-traumatic ONFH were directly associated with steroids^2^. Considering the serious consequences^3^ and economic costs^4^ incurred by ONFH, it is necessary to find new treatments. However, although numerous hypotheses for the pathogenesis of this disease, including lipid metabolism disorder, intravascular coagulation, microvascular injury, and intraosseous hypertension^5^,^6^,^7^,^8^, have been proposed, the exact mechanism of steroid-induced osteonecrosis of the femoral head (ONFH) still remains unclear. Yet, among all the risk factors reported, oxidative stress disorders may be one of the most common one participated^9^, which may benefit more when intervened.

Oxidative stress is defined as an imbalance between the production of oxidants (free radicals or reactive oxygen species, ROS) and their elimination by protective mechanisms, such as antioxidants^10^. ROS have been demonstrated to be closely related to ONFH^11^. Not only the wide influence oxidative stress disorders have in pulmonary, neurodegenerative, autoimmune diseases, as well as in metabolic disorders, cancer, and aging^12^,^13^,^14^. But also the direct evidence that chronic use of methylprednisolone may increase ROS damage^15^. In addition, it has been hypothesized that osteonecrosis is produced by the ischemic change accompanying the compartment pressure load in the marrow, where degeneration and necrosis may occur simultaneously with oxidative stress^16^. Besides, antioxidants such as Coenzyme Q10 (CoQ10)^17^ and hydrogen-rich saline^18^, have already been proved to be therapeutic for steroid-induced osteonecrosis in rats. While stem cell factor (SCF)^19^ and grape seed proanthocyanidin proved protective by reducing oxidative stress^20^. Based on the studies mentioned above, it is widely suspected that oxidative stress leads to osteonecrosis of ONFH^21^. Effective, selective, multifunctional drugs suppressing oxidative stress have tremendous potential in ONFH treatment, which can be realized by nanocomposites.

The choice of structural composition and compound method shall be very careful. As an essential trace mineral^22^, selenium (Se) plays an important role in suppressing oxidative stress^23^,^24^,^25^ and has been proved interrelationship with DNA damage and oxidative stress^26^. Se deficiency may induce oxidative stress and endoplasmic reticulum stress^27^ in some special cases (such as the use of ECMO)^28^. In contrast, Se’s essential interrelationship with coenzyme Q10 in cardiovascular diseases^29^ and its preventive effect in combination with vitamin E for prostate cancers^30^ suggested a great potential for the prevention of ONFH. Which made Se an ideal composition in ONFH treatment.

Compared with other reported ROS scavengers, Se is more stable and economical than a biological product. However, its toxicity cannot be ignored^31^ because Se has a narrow margin between beneficial and toxic effects^32^. After modification by nanotechnology, nano-Se can serve as an antioxidant with reduced risk of selenium toxicity^33^. The size of nanoparticles plays an important role in their biological activity: as expected, 5–200 nm Nano-Se can directly scavenge free radicals *in vitro* in a size-dependent fashion^34^. For further developments of nano-se therapy, porous structure of Se@SiO_2_ were synthesized. Our previous research showed that porous Se@SiO_2_ nanocomposites can be used as a delivery system for the controlled-release of Se nanoparticles^35^, which can slowly release a beneficial amount of Se over a long period of time. Moreover, Se nanocrystals exhibit a higher biological activity and lower toxicity than other forms^33^,^36^,^37^. Thus, porous Se@SiO_2_ nanocomposites may be a safe and ideal source of Se. Meanwhile, the porous structure Se@SiO_2_ nanocomposites have made them an ideal deliver system for multifunctional drug therapy which represents a promising, safe and effective way for ONFH treatments. In order to explain the mechanism porous Se@SiO_2_ nanocomposites have, the combination therapies are not considered in this study.

The following three aspects of porous Se@SiO_2_ nanocomposites were evaluated: the identification and biosecurity, the ROS-suppressing capacity and the therapeutic effects associated. For mechanism explain, experiments were repeated *in vitro* and *in vivo*.

## Experimental

### Synthesis and characterization of material

Porous Se@SiO_2_ nanocomposites were prepared according to our previous method^35^, and characterized by means of a D/max-2550 PC X-ray diffractometer (XRD; Rigaku, Cu-Kα radiation), a transmission electron microscopy (TEM; JEM-2100F). Besides the control release of Se were repeated at PH 7.4. As-synthesized porous Se@SiO_2_ nanocomposites were dispersed in deionized water for further use.

### Culture and identification of the cartilage cells

Two male SD rats, weighing 150 g, were sacrificed by CO_2_ asphyxiation and disinfected in 75% alcohol for 10 min. The head of the femur was exposed under aseptic conditions. Then, the total articular cartilage was isolated, collected, cut into 1 mm^3^ pieces, and then digested with 0.2% collagenase type II (GIBCO Grand Island, NY, USA) for 30 min, and filtered through a 70-μm cell strainer and washed 3 times with phosphate-buffered saline (PBS) at 300 × g for 5 min. After that, the collected cells were seeded into 100-mm tissue culture dishes and grown in DMEM/F-12 (GIBCO Grand Island, NY, USA) with 10% FBS (GIBCO Grand Island, NY, USA), 100 μg/ML streptomycin and 100 U/ML penicillin (GIBCO Grand Island, NY, USA) in a 37 °C, 5% CO_2_ environment. The culture medium was changed every 2–3 days. The cells were digested with 0.25% trypsin (GIBCO Grand Island, NY, USA) and passaged when they reached 80–90% confluency. Cells at passage 3 in culture were identified by immunofluorescence staining with collagenase type I (Santa Cruz, CA, USA) and aggrecan (Santa Cruz, CA, USA) before being used for subsequent experiments.

### Biocompatibility test and direct flow cytometry analysis

The cartilage cells were diluted into single cell suspensions and seeded into 96-well plates (1 × 10^4^ cells/well) with the culture medium. After 24 hours, the upper medium was exchanged with a medium containing different concentrations of porous Se@SiO_2_ nanocomposites that were dispersed by ultrasound. After additional 24 hours, a 10% cck-8 (DOJINDO, Japan) solution was added to each well, and the plates were incubated for 1 hour in the incubator. Then, the absorbance was measured at 490 nm using a micro-plate reader. Before a comparison, the absorbances were normalized to control groups without porous Se@SiO_2_ nanocomposites.

Before the flow cytometry analysis, the cartilage cells were cultured with the porous Se@SiO_2_ nanocomposites at a concentration of 40 μg/ML based on the results of the cck-8 assay for 24 hours. Then, the cartilage cells were digested and washed twice with blinding buffer (BD, USA) and analyzed directly on an Accuri C6 at the fluorescence channels for FITC, PE/PI, 7AAD and APC. For the apoptosis analysis, the cells were incubated with 5 μL of APC-Annexin V and 5 μL of PI for 15 min at room temperature (25 °C) in the dark, and then 400 μL of 1 × Binding Buffer was added to each tube. The cell suspension was then analyzed on an Accuri C6.

### Protection by the porous Se@SiO_2_ nanocomposites as assessed by suppression of ROS

H_2_O_2_ was considered a classical simulation of ROS^38^ and widely used^39^,^40^,^41^ which was also used in this experiment.

Cartilage cells were plated into 6-well plates (1 × 10^6^ cells/well) and were then pre-stimulated by the porous Se@SiO_2_ nanocomposites in a culture medium at a concentration of 0 μg/ML, 20 μg/ML, and 40 μg/ML for 24 hours, respectively. Subsequently, the cells in the plates and the EP tubes (collected by digestion for the flow cytometry test) were stimulated with 50 μM H_2_O_2_ (Sigma-Aldrich St. Louis, MO, USA) in PBS (1.5 mL/well, 500 μL/EP tube). 30 min later, DCFH-DA at its working concentration was added after 2 washes with PBS (1000 rpm, 5 min for intervention in EP tube). After 15 min of staining at 37 °C, the plates were directly observed with a fluorescence microscope (Leica, German). Cells in the EP tube were analyzed by flow cytometry.

For the cell supernatant tests, DF-12 (1.5 mL/well) was added to the 6-well plates after the H_2_O_2_ stimulation. IL-1β, IL-4 and IL-6 were assessed 24 hours later by Elisa Kits (Neobioscience, China).

### Protection by the porous Se@SiO_2_ nanocomposites as assessed by apoptosis tests and cell activity

For the TUNEL assay, the treatment group, with or without a 24-hour pre-stimulation with 40 μg/ML of the porous Se@SiO_2_ nanocomposites, was stimulated with 200 μM H_2_O_2_ in DF-12, while the control group was stimulated with DF-12 for 24 hours. The TUNEL assay was then used to assess DNA fragmentation by a commercially available kit (*In Situ* Cell Death Detection Kit, fluorescein, Roche, Indianapolis, IN, USA). Briefly, the fixed cells on the slides were washed three times for 5 min with PBS and permeabilized with 0.1% (v/v) Triton X-100 containing 0.1% (w/v) sodium citrate for 2 min. The samples were then incubated in 50 μL of TUNEL reaction mixture for 1 h at 37 °C in a dark and humidified atmosphere. Subsequently, 6-diamidino-2-phenylindole (DAPI) was used for staining of the nuclei. Positive TUNEL staining was observed under a fluorescence microscope. The sperm TUNEL index was evaluated by determining the ratio of the number of TUNEL-positive cells to that of total cells in each of the ten fields of vision.

For the flow cytometry analysis, cells received pre-stimulation or no stimulation with the porous Se@SiO_2_ nanocomposites in 6-well plates for 24 hours, and then 200 μM and 500 μM H_2_O_2_ in DF-12 were each used separately as inducements. After 24 hours, the suspension cells and adherent cells were collected and measured with an annexin V/APC apoptosis detection kit (eBioscience, USA). Briefly, the cells were trypsinized and pelleted by centrifugation, washed once with ice-cold PBS, and resuspended in 1 × Binding Buffer at a concentration of 1 × 10^6^ cells/ML, from which 100 μL of cell suspension (1 × 10^5^ cells) was transferred to a 1.5 mL EP tube. Staining was then completed that is outlined above.

For the cck-8 assay, the cartilage cells were diluted into single cell suspensions and seeded into 96-well plates (1 × 10^4^cells/well) with a culture medium. After 24 hours, the upper medium of the experiment group was exchanged with a medium with 40 μg/mL of the porous Se@SiO_2_ nanocomposites, while the control group had a replacement of a medium without the porous Se@SiO_2_ nanocomposites. After a 24-hour stimulation, different concentrations of H_2_O_2_ were used for stimulation. After additional 24 hours, a 10% cck-8 (DOJINDO, Japan) solution was added to each well, and the plates were incubated for 1–2 hours in the incubator. Then, the absorbance was measured at 490 nm using a micro-plate reader.

## Animal experiments

### Animal preparation

This study was performed following the National Institutes of Health guidelines for the use of experimental animals, and all animal protocols were approved by the Institutional Animal Care and Use Committee of Shanghai Jiaotong University. Male Sprague-Dawley (SD) rats (weight 250–300 g; age of 12 months; SPF class) were obtained from the experimental animal center of Shanghai Jiao Tong University. The rats were bred and maintained under a 12/12-hour light-dark cycle with free access to food and water. The room temperature was set to 18 °C–25 °C, and the relative humidity was set to 40–60%.

### Testing changes in ROS levels in serum after stimulation by the porous Se@SiO_2_ nanocomposites *in vivo*

Prior to the experiments on the ONFH model, the porous Se@SiO_2_ nanocomposites were directly injected into adult rats intraperitoneally at doses of 0 mg/kg, 1 mg/kg, 2 mg/kg and 4 mg/kg. 24 hours later, the serum from each group was cultured as outlined in a previous study^42^, and the ROS (Nanjing Jiancheng, China) was measured with an Elisa kit.

### ONFH animal model establishment, treatments and sample collection

According to a previous study^43^, an early stage SANFH model was induced using a combination of lipopolysaccharides (LPS) and methylprednisolone (MPS). 36 rats were randomly divided into a control group (group A), model group (group B) and porous Se@SiO_2_ nanocomposite group (group C), each consisting of 12 rats. Male SD rats from group B and group C were intravenously injected with LPS (10 μg/kg body weight). After 24 hours, three injections of MPS (20 mg/kg body weight) were administered intramuscularly every 24 h for 5 days. To prevent infection, each rat was intramuscularly injected with 100,000 U of penicillin. Based on the toxicity study data of selenium nanoparticles in rats^37^ and control release capacity of porous Se@SiO_2_ nanocomposite^35^. The rats in group C were injected intraperitoneally with 1 mg/kg of the porous Se@SiO_2_ nanocomposites per day for 14 days, beginning 4 weeks after the MPS administration. The model group (group A) was fed and housed under identical conditions but received saline injections.

The rats in all groups were sacrificed by an overdose of anesthesia at 8 weeks after the first MPS injection, and the femoral heads and blood samples were harvested. Blood samples from all groups were collected in containers without anticoagulant, thus allowing clot formation. The blood was centrifuged at 1,200× g for 10 minutes. The serum was stored at −80 °C until further analysis. The left femoral heads of all rats were preserved in a −70 °C cryogenic freezer immediately after sacrifice, and the proteins were isolated for Western blot analysis. The right femoral heads were collected and immediately fixed with 10% formalin (0.1 M phosphate buffer, pH 7.4) at 4 °C for 24 hours. Then, the samples were used for Micro CT scanning and HE staining tests following previous protocols^44^.

### Evaluation techniques

#### Micro CT procedure

According to a previous study^44^, a Micro CT (GE Healthcare Biosciences, Piscataway, NJ, USA) was used to detect changes in the excised femoral head sample and the trabecular bone. Bone volume (BV), bone surface (BS), trabecular thickness (Tb.N), trabecular number (Tb.Sp) and trabecular separation (Tb.Th) were calculated.

#### Western blot analysis

The protein expression levels of IL-1β^45^, collagen II^46^, MMP-13^47^ and aggrecan^48^ in the femoral head tissues obtained from rats in the different groups were detected by Western blot analysis. The Western blot protocol and semi-quantitative analysis were carried out following the protocols of a previous study^43^. Antibodies, obtained from Santa Cruz Biotechnology (Santa Cruz, CA, USA), against the following were used: IL-1β, collagen type II, MMP-13, aggrecan, and GAPDH.

#### Hematoxylin-eosin staining

According to a previous study^49^, the femoral heads harvested from the animals were fixed with 4% formalin and were then washed with PBS buffer. Sequentially, they were decalcified with 10% EDTA and neutralized with sodium sulfate buffer for approximately 4 weeks. After decalcification, the tissues were embedded in paraffin and cut in the coronal plane into 4-μm thick sections with a microtome. Then, H-E staining was processed for the micro-structure observation.

#### Serum Biochemistry

Serum from each groups were tested. The activities of blood serum marker enzymes, such as alanine transaminase (ALT), alkaline phosphatase (ALP), aspartate transaminase (AST), creatinine, and urea, were measured using a Roche kit (Penzberg, Germany) and analyzed spectrophotometrically using the Hitachi Analytical Instrument (Roche Diagnostic GmbH, Mannheim, Germany).

### Statistical analysis

Quantitative data are expressed as the mean ± SD. Data were analyzed using SPSS 21.0 software (IBM, Armonk, NY, USA). For comparisons of means among multiple groups, one-way ANOVAs followed by LSD tests was performed. Differences were considered statistically significant when P < 0.05.

## Results

**Figure 1:**
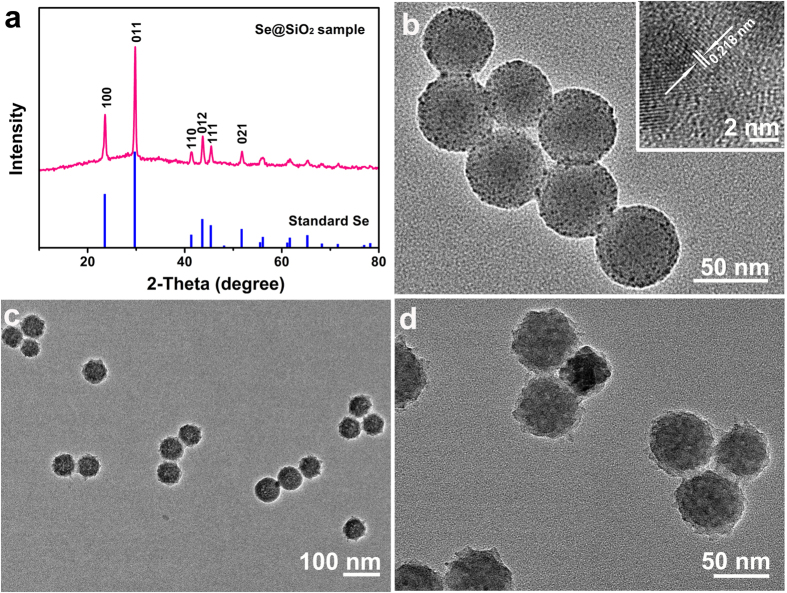
The characterization of the Se@SiO_2_ nanocomposites: (**a**) XRD pattern of the Se@SiO_2_ nanocomposites and the standard hexagonal phase of Se (JCPDS card no: 65-1876). (**b**) TEM image of as-prepared Se@SiO_2_ nanocomposites (inset: HRTEM of a Se@SiO_2_ nanocomposite). (**c**) Low magnification and (**d**) high magnification of the porous Se@SiO_2_ nanocomposites.

A series of tests demonstrated that the nanocomposites used in the following experiments were the porous Se@SiO_2_ nanocomposites. The phase structure of the resulting nanocomposites was examined by the XRD pattern, as shown in Fig. 1a. Several well-defined characteristic peaks, such as (100), (011), (110) and (012), exhibited the hexagonal phase, referenced by the standard Se phase (JCPDS card no. 65-1876). In addition, the XRD pattern of the Se@SiO_2_ nanocomposites showed a steady increase in the low angle region, which is due to amorphous silica. It can be observed in Fig. 1b that the homogeneous nanocomposites have a diameter of about 55 nm, which many very small nanoparticles (less than 5 nm) were interspersed from the center to the surface. The dispersed nanoparticles have an interplanar spacing of 0.218 nm, matching the spacing for the (110) crystal planes of the standard hexagonal Se (Fig. 1b inset), which further confirmed that the small nanoparticles dispersed in the silica were Se nanocrystals. After treatment with hot water, the porous Se@SiO_2_ nanocomposites were formed (Fig. 1c,d). Our previous characterization of BET showed that the Se@SiO_2_ nanocomposites are porous^35^. The PVP (pyrrolidinovalerophenone) had permeated into the silica shells, and the channels were distributed in the Se@SiO_2_ nanocomposites, leading to the slow release of very small nanoparticles from the porous Se@SiO_2_ nanocomposites. Meanwhile, the release capacity were re-confirmed (Fig. S1).

**Figure 2:**
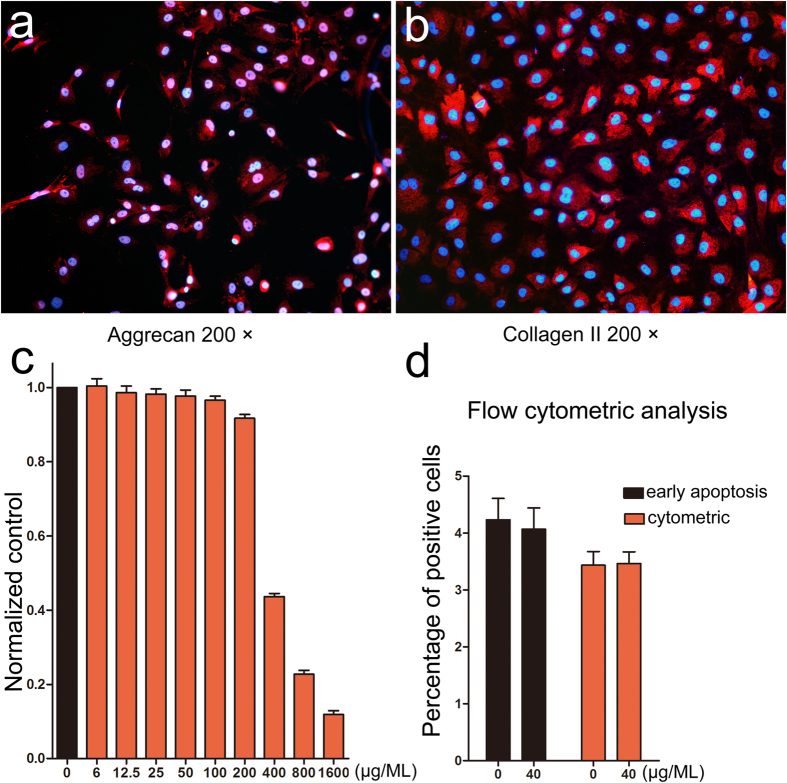
Identification of cartilage cells and confirmation of biosafety of the porous Se@SiO_2_ nanocomposites: (**a**) Immunofluorescence staining of aggrecan (200×). (**b**) Immunofluorescence staining of collagenase type I (200×). (**c**) Normalized cck-8 absorbance at 490 nm of cells cultured with different porous Se@SiO_2_ nanocomposite concentrations. Obviously toxicity only occurs over 100 μg/ML. (**d**) Flow cytometry of cartilage cells stimulated with 40 μg/ML of the porous Se@SiO_2_ nanocomposites for 24 hours. No significant differences indicated (P > 0.05).

Cells were identified as cartilage cells by staining with aggrecan and collagenase type I (Fig. 2a,b). Co-culturing with the porous Se@SiO_2_ nanocomposites was demonstrated to have no significant influence on the flow cytometry (Figs S2 and S3) and proved to be safe by flow cytometry at a concentration of 40 μg/ML (Figs 2c, S4). The porous Se@SiO_2_ nanocomposites showed no significant cytostatic action under a concentration of 40 μg/ML compared with the control group (Fig. 2d).

**Figure 3:**
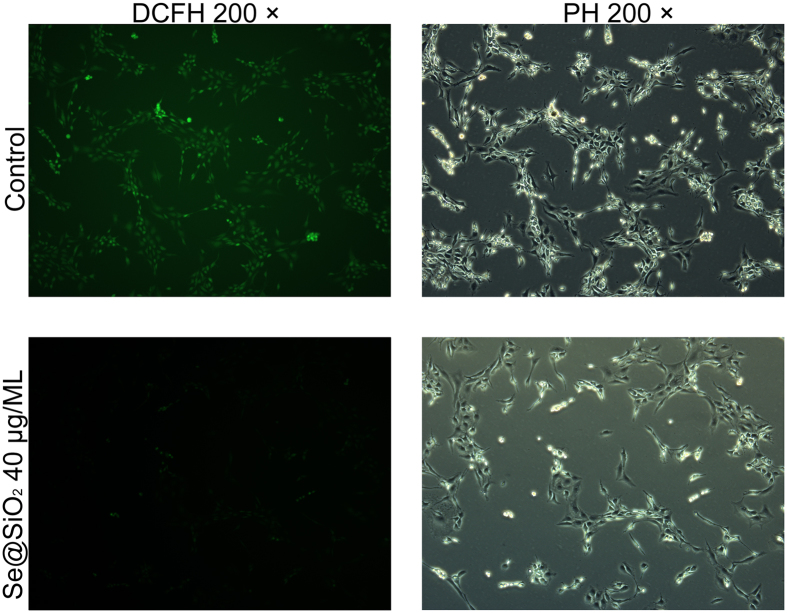
Suppression of ROS function as indicated by DCFH staining after a 30-min stimulation with 50 μM H_2_O_2_ diluted in PBS. DCFH staining was used for ROS detection in cartilage cells pre-stimulated with and without the porous Se@SiO_2_ nanocompositesv (40 μg/ML for 24 hours pre-stimulation). ROS expression was indicated by the fluorescence intensity of DCFH (FITC). Pre-stimulated with the porous Se@SiO_2_ nanocomposites can significantly decrease ROS expression.

The fluorescence microscopic observation indicated that pre-stimulation with the porous Se@SiO_2_ nanocomposites might significantly decrease the expression of the ROS (Fig. 3) after H_2_O_2_ exposure, which was re-confirmed and quantitatively analyzed by flow cytometry analysis (Fig. S5). Pre-stimulation with the porous Se@SiO_2_ nanocomposites could significantly reduce the expression of IL-1β, IL-4 and IL-6 in a concentration-dependent manner (Fig. S6, P < 0.05).

**Figure 4:**
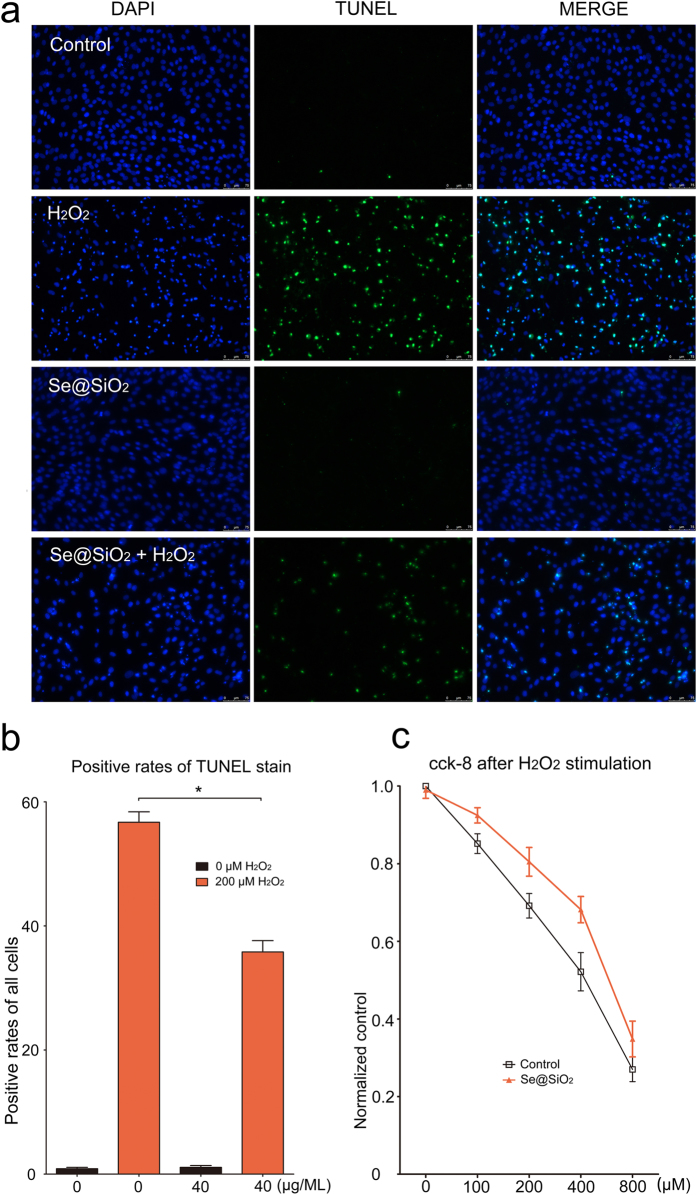
(**a**) Staining of cartilage cells with DAPI, TUNEL and MERGE. (**b**) Statistical graph and analysis of TUNEL staining, Pre-stimulated with the porous Se@SiO_2_ nanocomposites can significantly decrease apoptosis rates induced by H_2_O_2_. *P < 0.05. (**c**) Normalized by control group the cell activity decreased along with the increasing H_2_O_2_ concentrations. Significant protection of the porous Se@SiO_2_ nanocomposites as reflected by the cck-8 assay P < 0.05.

After 24 hours of stimulation with 200 μM H_2_O_2_, the cells pre-stimulated with 40 μg/ML of the porous Se@SiO_2_ nanocomposites demonstrated less damage than the control groups. The apoptosis analysis showed that the porous Se@SiO_2_ nanocomposites significantly decreased the apoptosis rates caused by H_2_O_2_ (Fig. 4a,b, *P < 0.05), which was re-confirmed by the flow cytometry analysis (Fig. S7, *P < 0.05) and the cck-8 assay (Fig. 4c, *P < 0.05) at different H_2_O_2_ concentrations.

After 24 hours through the intraperitoneal injection of the porous Se@SiO_2_ nanocomposites, the ROS levels in the serum were significantly decreased (Fig. S8, *P < 0.05 in comparison with the other groups). 8 weeks after the steroid injection, the weight of the rats was maintained at 426 ± 32 g, with no significant differences detected among the groups.

The porous Se@SiO_2_ nanocomposite treatment was performed for 4 weeks after the steroid exposure to avoid the possible influence of the model inducement. Under this situation, the protective role of the porous Se@SiO_2_ nanocomposites in the early stage of ONFH were evaluated.

**Figure 5:**
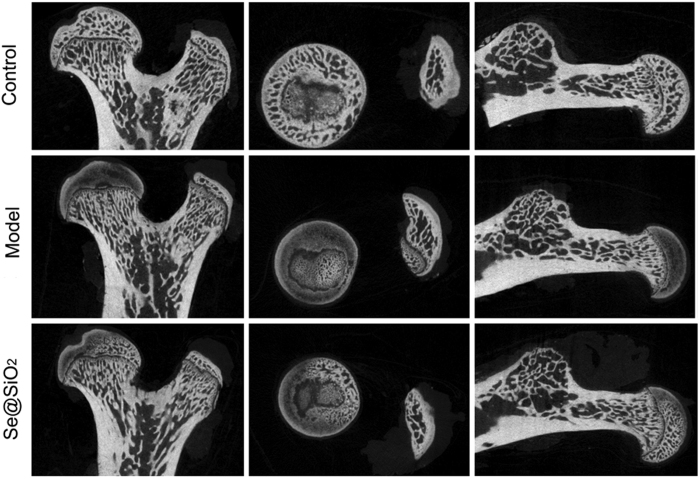
Two-dimensional pictures of normal bone from normal rats, and osteonecrotic bones from steroid-induced ONFH rats with or without the treatment by the Porous Se@SiO_2_ nanocomposites. The necrosis area (region of low-density) significantly reduced by Porous Se@SiO_2_ nanocomposites therapy, yet not completely cured referring to control.

A CT scan indicated the therapeutic effects of the Porous Se@SiO_2_nanocomposite treatment (Fig. 5), and the data analyses on the levels of the microstructural parameters showed significant differences (Fig. S9).

Serum ALT, ALP, AST, creatinine, and urea in each group had no significant differences (Table S1).

**Figure 6:**
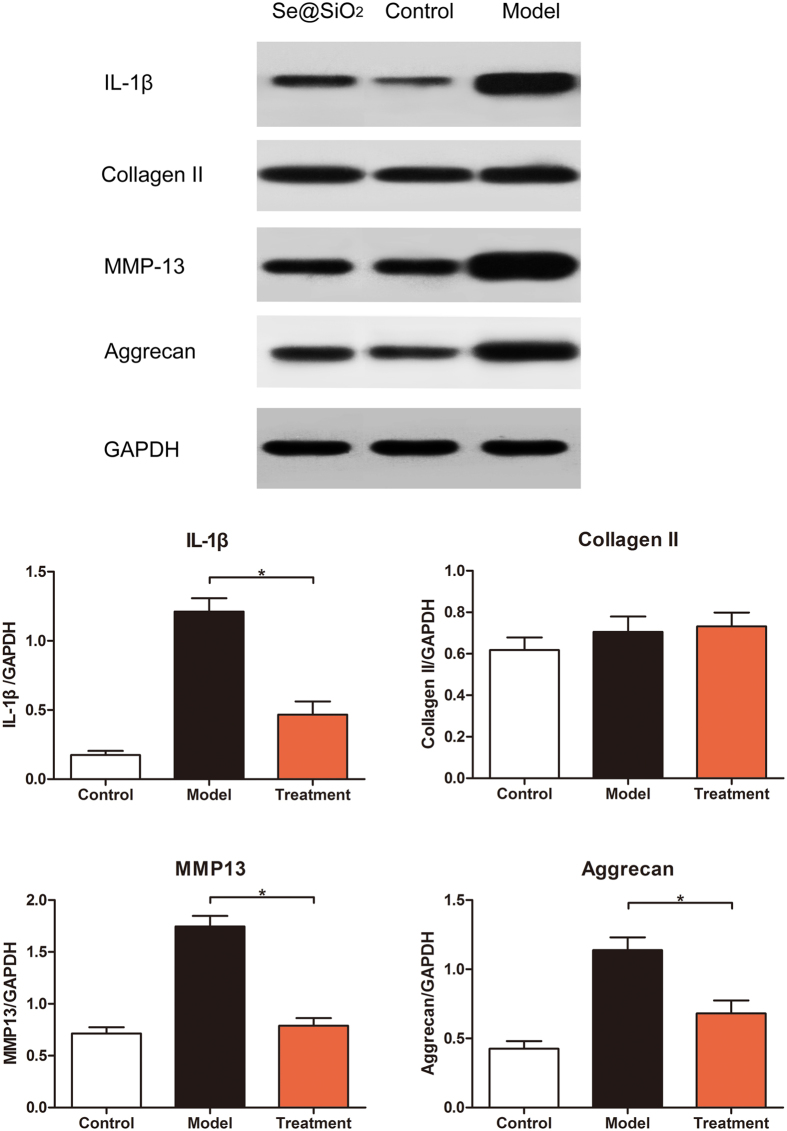
Western blot analysis of IL-1β, collagen type II, MMP-13, aggrecan, and GAPDH expression of cartilage in the control group and groups.

**Figure 7:**
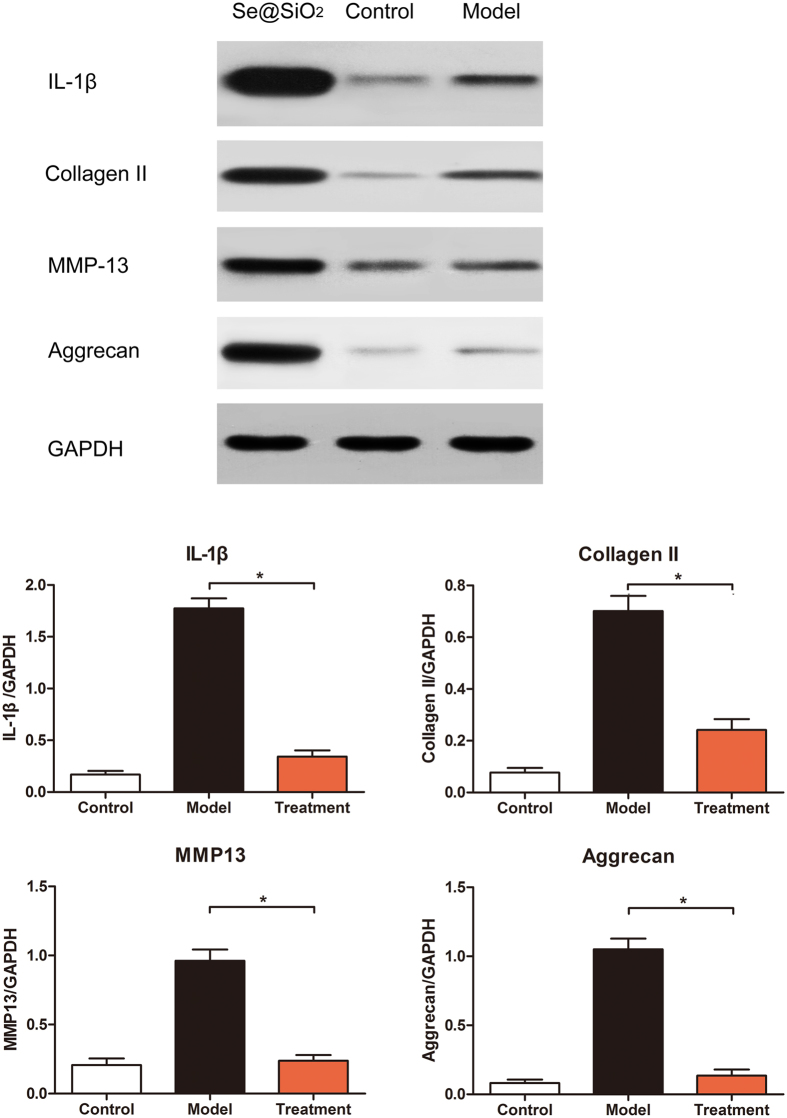
Western blot analysis of IL-1β, collagen type II, MMP-13, aggrecan, and GAPDH expression of subchondral bone in the control group and the groups.

IL-1β and MMP-13 were classical damage indexes in cartilage reaches that can objectively evaluate the necrosis situation. While collagen type II and aggrecan were both constructional and functional protein in cartilage. The changes of these four protein assessed by Western blot analysis can fully reflect the specific situation. Western blot analysis for both subchondral bone and cartilage showed that the expression of IL-1β, collagen type II, MMP-13 and aggrecan increased in the model group. After the Porous Se@SiO_2_nanocomposite injection, expression significantly decreased (Figs 6 and 7, P < 0.05), except for collagen II expression in cartilage (Fig. 6). The blot is representative of subchondral bone, normalized to GAPDH, and statistical analysis was performed (n = 12, *P < 0.05) (b, d); the blot is representative of cartilage, normalized to GAPDH, and statistical analysis was performed (n = 12, *P < 0.05).

**Figure 8:**
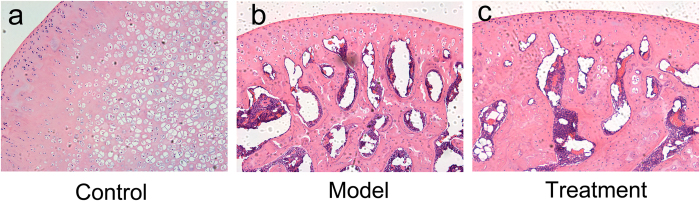
Histological images (40×) demonstrating methylprednisolone-induced femoral head necrosis (**b**) compared with the control group (**a**). After the Porous Se@SiO_2_ nanocomposite injection, the destruction of the bone structure was obviously alleviated (**c**).

Hematoxylin-eosin staining were used for structural observation of the femur head. Serious destruction of the femur head was induced for 8 weeks after methylprednisolone administration. After the Porous Se@SiO_2_ nanocomposite injection, fewer necrotic areas (cavity beneath the cartilage surface) were detected (Fig. 8).

## Discussion

Briefly, after Porous Se@SiO_2_ pre-stimulation before H_2_O_2_ exposure, ROS level were significantly decreased (Figs 3, S5), thus IL-1β, IL-4 and IL-6 levels were tested lower by Elisa (Fig. S6) and apoptosis rates decreased according to TUNEL staining (Fig. 4b) and flow cytometry analysis (Fig. S7). The cell activity were protected (Fig. 4c). *In vivo*, ONFH rat models were successfully established and Porous Se@SiO_2_ nanocomposites proved safe (Table S1) and effective by Micro CT scanning (Figs 5, S9), serum Elisa assay (Fig. S8), Western blotting (Figs 6 and 7) and HE staining (Fig. 8). The Porous Se@SiO_2_ nanocomposites proved medicative to steroid-induced ONFH by reducing oxidative stress.

Still, the mechanism of ONFH are still unclear, but ROS and ROS suppressing treatments indicated the close relationship oxidative stress have to ONFH. A newly reported review of hyperbaric oxygen therapy in the treatment of osteonecrosis of the femoral head confirmed the therapeutic function antioxidants have in ONFH^50^. Thus by decreasing the ROS levels, other elements should also have the capacity to retard the progress of ONFH. However, antioxidants, such as coenzyme Q10 (CoQ10), may lead to nausea, upset stomach or loss of appetite. In addition, hydrogen-rich saline is not stable enough, and stem cell factor (SCF) and grape seed proanthocyanidin are costly as standard treatments. Compare to which, thanks to the SiO_2_-coated structure, significant advantages Porous Se@SiO_2_ nanocomposites have in both economic and stability (room temperature store) are convinced.

Moreover, porous structure gave Se@SiO_2_ nanocomposites potentials to be multifunctional (combined with drugs) and made them slow-released. Controlled-release systems play special roles in disease treatment^51^,^52^. Compared to normal Se nanoparticles, Se in the Se@SiO_2_ nanocomposites is limited by SiO_2_. Accompanying the entrance of PVP into an aqueous solution, trace Se can be released into the solution^35^. By controlled-release, the porous Se@SiO_2_ nanocomposites may have advantages in biosafety (Fig. 2d) and *in vivo* stability. Because the process of ONFH is long and progressive^53^, the controlled-release capacity benefits the sustaining of the ROS inhibition. So, SiO_2_-coated ultrasmall Se particles may help to delay the onset or reduce the serious outcome of the ONFH.

The advantages and characteristics porous Se@SiO_2_ nanocomposites have made them an ideal therapy to ONFH. The possible mechanism may be the ROS suppressing. Nano-materials that can mediate the ROS expression are not unique; some function by direct contact, and some function by anti-bacterial properties^54^, while others help to maintain structural stability and improve bio-safety^55^, even help to induce the cell apoptosis program via the ROS^56^. However, the porous Se@SiO_2_ nanocomposites may not only reduce the expression of the ROS directly but also provide essential elements that help to comprise the intracellular pool against oxidative stress^57^,^58^. Accumulating evidence supports the idea that Se nanoparticles have antioxidant effects^59^. These effects have been shown to increase the activities of both GPX and glutathione S-transferase and induce less oxidative stress^34^,^59^. The same consequences were observed in this study, in which the porous Se@SiO_2_ nanocomposite simulation significantly decreased the ROS levels and improved the cells’ tolerance to H_2_O_2_ (Fig. 4), with the ROS levels in serum demonstrating the same consequence *in vivo* (Fig. S7). It also has been reported that by inhibiting the activation of the PI3K/AKT and ERK signaling pathways and endoplasmic reticulum stress, Se can suppress oxidative-stress-enhanced vascular smooth muscle cell calcification^60^, further reducing the levels of IL-1β, TNF- α, oxidative stress, and NF- κ B activation^61^. On the contrary, a diet with a Se deficiency weakens antioxidant capacity^62^. However, despite the acute side effects associated with toxicity^63^, pre-stimulation with porous Se@SiO_2_ nanocomposites did help to reduce the expression of IL-1β, IL-4, and IL-6 after exposure to H_2_O_2_ in a concentration-dependent manner (Fig. S5). This change matched the decreased IL-1β level in the femoral head (Figs 6 and 7).

This improvement was reflected not only by Micro CT (Fig. 5) and the histological images (Fig. 8) but also by the protein levels of tissue biopsies (Figs 6 and 7). In the CT imaging, a low attenuation section in the femoral head refers to collapse and necrosis. Observed in 3-dimensional images, after treatment by the porous Se@SiO_2_ nanocomposites, the percentage of low attenuation area was reduced. By a quantitative comparison (Fig. S8), significant differences were detected among these groups confirmed the curative effects. Meanwhile, the tissue biopsy directly showed the improvement in the femur head structure in treatment group (Fig. 8). Thus, it is receivable that after porous Se@SiO_2_ nanocomposite treatment, anatomic integrity of femoral head was maintained.

Western blot investigation showed a significant increase in IL-1β and MMP-13, which indicates that damage had occurred in the cartilage and subchondral bone, which decreased after the porous Se@SiO_2_ nanocomposite treatment. The expression of aggrecan and collagen type II indicated a compositional variation after steroid inducement, as their variation combined the structural and functional changes in the femoral head, which were also significantly recovered after the porous Se@SiO_2_ nanocomposite treatment. These results confirmed the local phenomenon and possible mechanism that ONFH may started with IL-1β and MMP-13 damages, influenced the structural and functional protein aggrecan and collagen type II and finally leads to necrosis (Fig. 6). By suppressing IL-1β and MMP-13, porous Se@SiO_2_ nanocomposites can significantly reduce the damage of ONFH.

However, in this experiment with the porous Se@SiO_2_ nanocomposites injection, only its possible function in the early stage of ONFH was tested, and the potential preventive effects of the porous Se@SiO_2_ nanocomposites, according to the basic function of ROS inhibition, were not demonstrated. For preventive treatment, it has to be proved that the porous Se@SiO_2_ nanocomposites have no influence on the therapeutic effects of the steroids. Another study system will be needed and will be reported in the future.

## Conclusions

In summary, the porous Se@SiO_2_ nanocomposites could reduce the ROS levels, protect cells from H_2_O_2-_induced apoptosis and reduce inflammation by inhibiting oxidative stress. Same effects were observed *in vivo* and eventually demonstrated the significant protective effect in disease management.

The porous Se@SiO_2_ nanocomposites have advantages of stability, economy, and enormous multifunctional potentials compared to other methods. Therefore, make porous Se@SiO_2_ nanocomposites an ideal way to protect the femoral head from osteonecrosis after steroid inducement.

## Additional Information

**How to cite this article:** Deng, G. *et al*. Treatment of steroid-induced osteonecrosis of the femoral head using porous Se@SiO_2_ nanocomposites to suppress reactive oxygen species. *Sci. Rep.* **7**, 43914; doi: 10.1038/srep43914 (2017).

**Publisher's note:** Springer Nature remains neutral with regard to jurisdictional claims in published maps and institutional affiliations.

## Supplementary Material

Supportiong Information
