# Supplementary material for: Treatment of steroid-induced osteonecrosis of the femoral head using porous Se@SiO2 nanocomposites to suppress reactive oxygen species
Source: Sci Rep. 2017 Mar 3;7:43914. doi: 10.1038/srep43914 (PMC5335566; doi:10.1038/srep43914)
Supplement: Supportiong Information [file srep43914-s1.pdf]

Electronic Supplementary Information

Treatment of steroid-induced osteonecrosis of the femoral head using porous Se@SiO_2 nanocomposites to suppress reactive oxygen species

Guoying Deng[‡,a], Kerun Niu[‡,b], Feng Zhou[b], Buxiao Li[a], Yingjie Kang[f], Xijian Liu[d], Junqing Hu[e], Bo Li[g], Qiugen Wang[a] Chengqing Yi[*,c], and Qian Wang[*,a,e]

a. Trauma Center, Shanghai General Hospital, Shanghai Jiaotong University School of Medicine, 650 Xin Songjiang Road, Shanghai 201620. P.R.China.

b. Department of Orthopedics, Shanghai Bone Tumor Institute, Shanghai General Hospital of Nanjing Medical University, Shanghai 200080, P.R.China.

c. Department of Orthopedics, Shanghai Bone Tumor Institute, Shanghai General Hospital, Shanghai Jiao Tong University School of Medicine, No. 100 Haining Road, Shanghai 200080, P.R.China.

d. College of Chemistry and Chemical Engineering, Shanghai University of Engineering Science, Shanghai, 201620, P.R.China.

e. State Key Laboratory for Modification of Chemical Fibers and Polymer Materials, College of Materials Science and Engineering, Donghua University, Shanghai 201620, P.R.China.

f. Department of Radiology, Shuguang Hospital, Shanghai University of Traditional Chinese Medicine. No. 528, Zhangheng Road, Shanghai 201203, P.R.China.

g. State Key Laboratory of High Performance Ceramics and Superfine Microstructure, Shanghai Institute of Ceramics, Chinese Academy of Sciences,

No. 1295 Dingxi Road, Shanghai 200050, People's Republic of China.

**Corresponding Authors: Qian Wang (e-mail: drwangqian23@163.com), Chengqing Yi (e-mail: ycq3000@126.com)

‡ These authors have contributed equally.

1. Supplementary Figures:

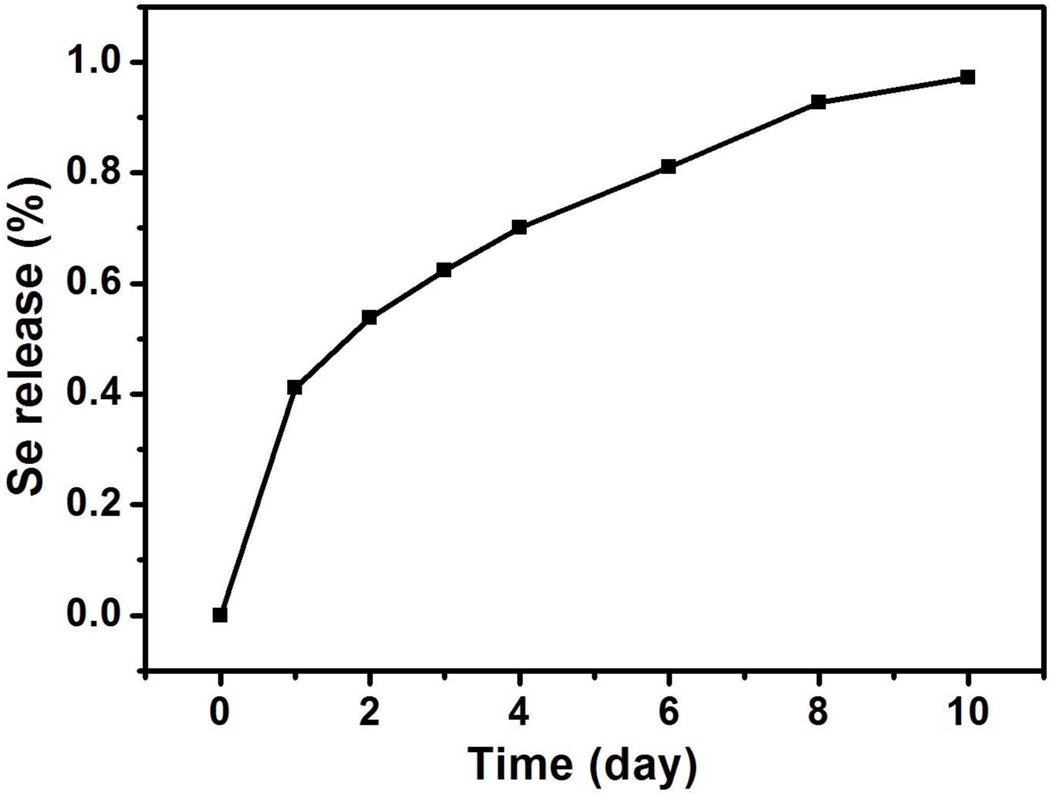

Figure S1 Control release capacity of porous Se@SiO_2 nanocomposites in PBS(pH=7.4)

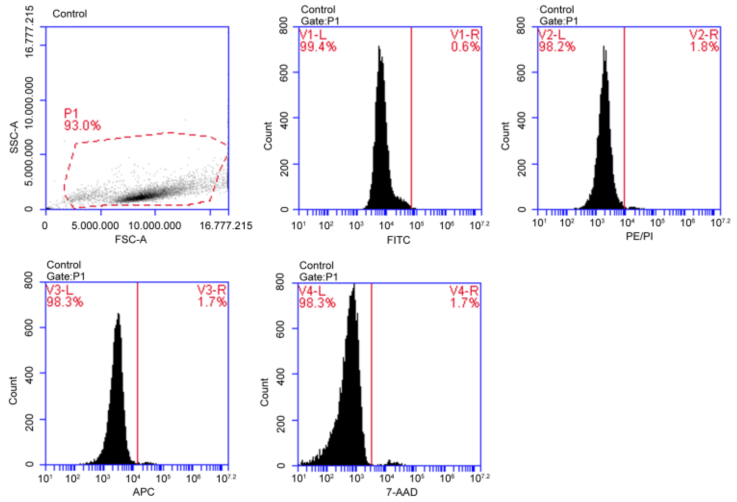

Figure S2 Flow cytometry of cartilage cells without porous Se@SiO_2 nanocomposites. The fluorescence intensity of FITC, PE/PI, 7AAD and APC have been tested.

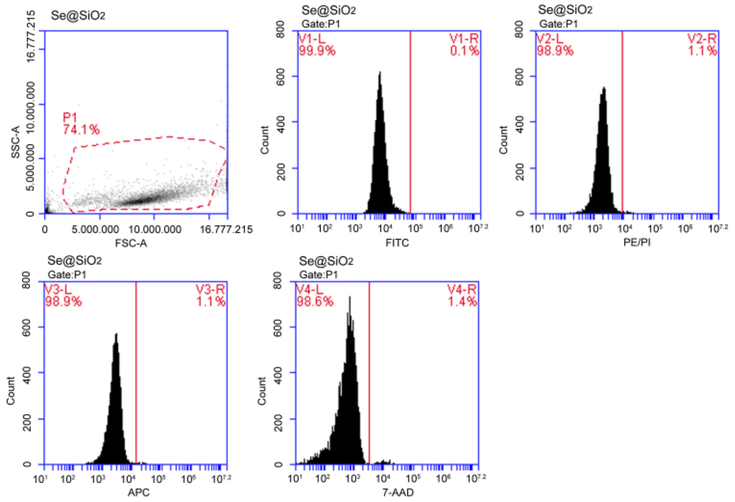

Figure S3 Flow cytometry of cartilage cells stimulated with 40μg/ML porous Se@SiO_2 nanocomposites. After stimulation, the fluorescence intensity of FITC, PE/PI, 7AAD and APC had no significant change (Fig. S1).

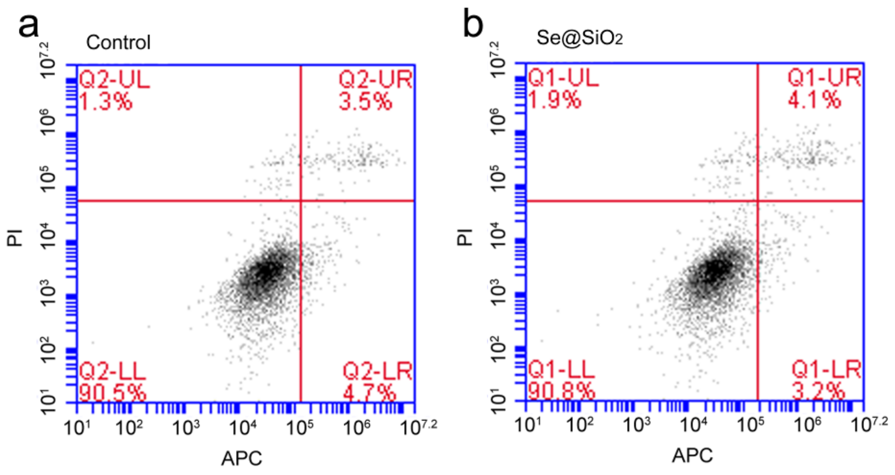

Figure S4 Flow cytometry of cartilage cells of annexin V APC and PI. The apoptosis rates of cartilage cells stimulated by 40μg/ML porous Se@SiO_2 nanocomposites (b) had no significant difference to control (a) in apoptosis rates.

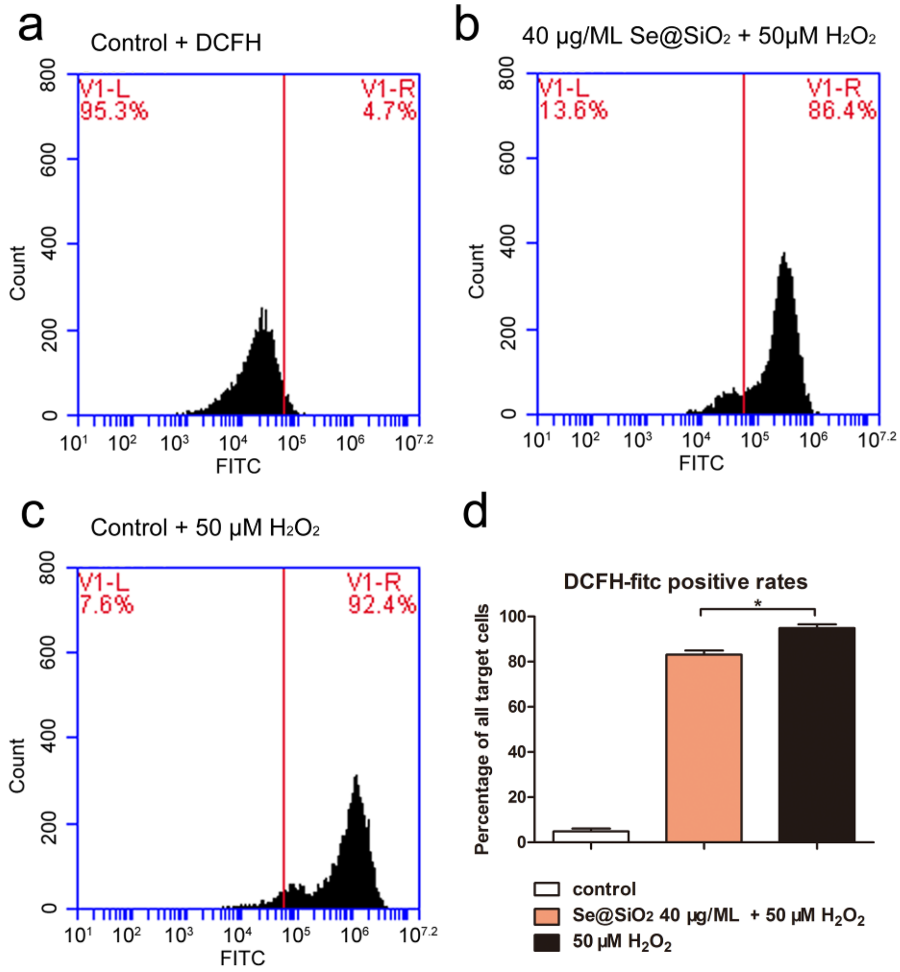

Figure S5 (a) FITC intensity of cartilage cells without porous $Se@SiO_2$ nanocomposites, DCFH-DA added without H_2O_2 stimulation. (b) FITC intensity of cartilage cells pre-stimulated with 40μg/ML porous $Se@SiO_2$ nanocomposites, DCFH assay after 50μM H_2O_2 stimulation for 15 min. (c) FITC intensity of cartilage cells without pre-stimulation, DCFH assay after 50μM H_2O_2 stimulation for 15 min. (d) FITC intensity significantly decreased by porous $Se@SiO_2$ nanocomposites stimulation. Statistical analysis $P < 0.05$

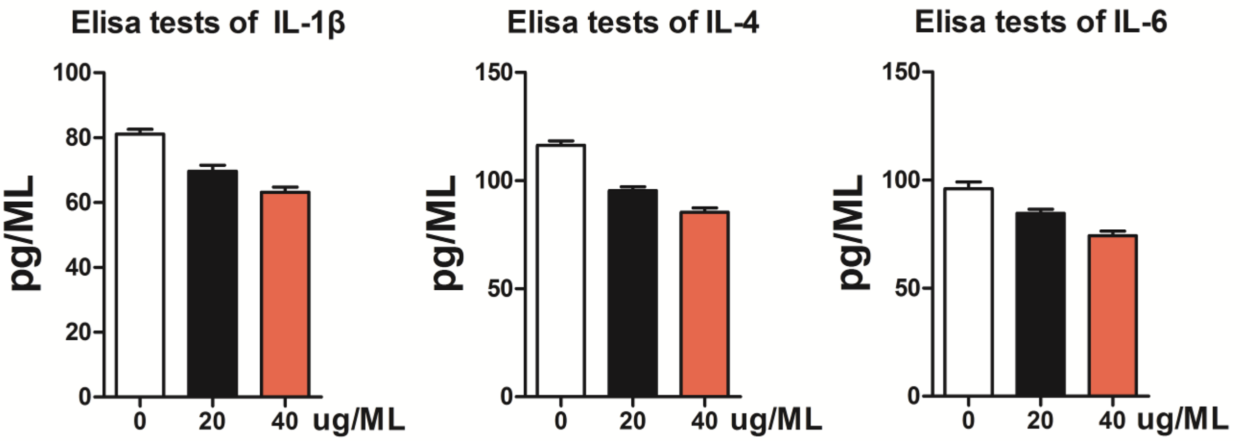

Figure S6 After pre-stimulated by different concentration of porous $Se@SiO_2$ nanocomposites for 24 hours, cells were all stimulated by 50μM H_2O_2. Concentrations of IL-1β, IL-4 and IL-6 in supernatant all have significant differences between each groups (*$P<0.05$).

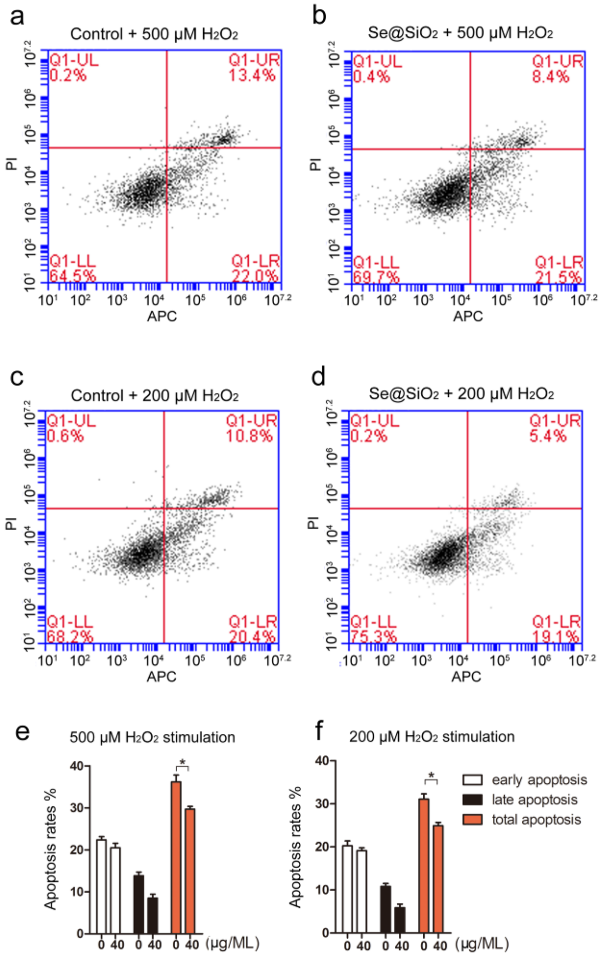

Figure S7 Apoptosis analysis of flow cytometer analysis. Both in H_2O_2 concentration of 500μM and 200μM, cells pre-stimulated by 40ng/ML porous Se@SiO_2 nanocomposites (b, d) suffered lower apoptosis rates than control groups (a, c) The statistic results showed significant differences that porous Se@SiO_2 nanocomposites pre-stimulation can decrease the apoptosis rates caused by H_2O_2. (e, f) *$P<0.05$

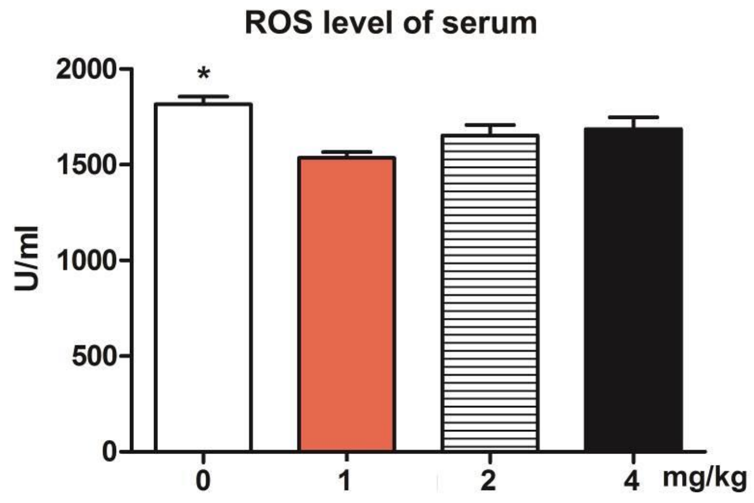

Figure S8 ROS level of serum 24 hours after porous Se@SiO_2 nanocomposites injection.

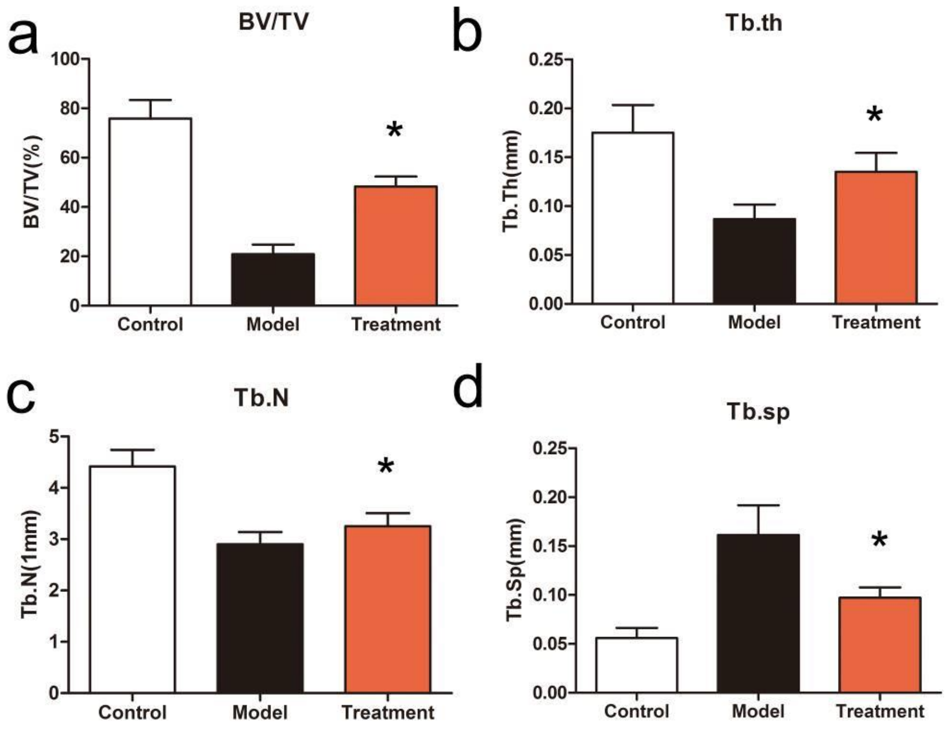

Figure S9 Quantitative analysis of CT scan: (a) the bone volume/tissue volume (BV/TV), (b) trabecular thickness (Tb.Th), (c) trabecular number (Tb.N), were significantly reduced, while (d) the trabecular separation (Tb.Sp) was significantly increased in rats with steroid-induced ONFH when compared with controls. Porous Se@SiO_2 nanocomposites can help reduced the structural

damages caused by methylprednisolone. Groups all have significant differences from each other (P<0.05).

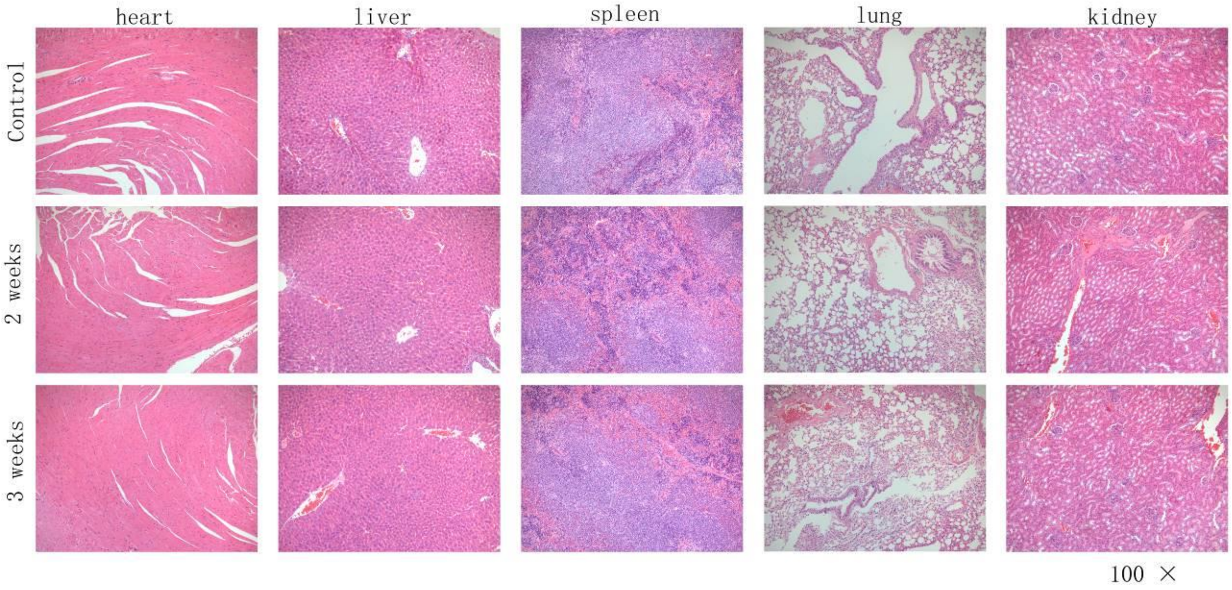

Figure S10 HE-staining of heart, liver, spleen, lung and kidney samples of healthy control and rats injected with Porous Se@SiO_2 nanocomposites. (100 ×)

Table S1

	ALT (U/L)	ALP (U/L)	AST (U/L)	Creatinine (umol/L)	Urea (mmol/L)
Normal	48.81±6.82	88.42±23.14	145.28±25.32	74.21±15.26	7.34±1.75
Control	49.20±5.91	87.49±24.02	144.65±25.32	72.56±13.26	7.82±1.95
Se@SiO_2	51.12±7.62	90.27±26.21	148.06±22.32	73.21±16.26	8.10±2.10

ALT: alanine transaminase; ALP: alkaline phosphatase; AST: aspartate transaminase.

Values are expressed as mean ± SD (n = 6). Values in the same column with different superscript letter indicate significant difference by one-way ANOVAs followed by LSD tests (P ＞ 0.05).
